# Mild Cognitive Impairment Recovery:Role of Resisting Ageism and Embracing Positive Age Beliefs

**DOI:** 10.1093/geroni/igaf122.919

**Published:** 2025-12-31

**Authors:** Becca Levy, Martin Slade

**Affiliations:** Yale School of Public Health, New Haven, Connecticut, United States; Yale University, New Haven, Connecticut, United States

## Abstract

It is widely assumed that individuals who develop mild cognitive impairment (MCI) will not recover. Yet nearly half of older persons with MCI regain normal cognition. The reason for this improvement is not well understood. This study is the first to consider whether a culture-based factor—positive age beliefs—contributes to MCI recovery. In previous experimental studies with older persons, positive age beliefs reduced stress caused by cognitive challenges, increased self-confidence about cognition, and improved cognitive performance. We therefore hypothesized that older individuals with positive age beliefs would demonstrate higher and faster rates of cognitive recovery compared to those with negative age beliefs. Data from the Health and Retirement Study provided a cohort of 1716 participants, aged 65 and older, all of whom had baseline MCI. As predicted, participants with positive age beliefs had a 30.2% higher likelihood of cognitive recovery and transitioned from MCI to normal cognition more rapidly (hazard ratio, 1.26; 95% CI, 1.08-1.46; p = .003). Additionally, those with positive age beliefs showed lower rates of developing MCI over a 12-year period. These results underscore the potential of addressing ageism by promoting positive age beliefs at both individual and societal levels, suggesting that such interventions could enhance cognitive recovery and reduce MCI prevalence.

